# Editorial: Insights in ethnopharmacology: 2021

**DOI:** 10.3389/fphar.2022.997577

**Published:** 2022-09-20

**Authors:** Cheorl-Ho Kim, Valentina Echeverria Moran, Judit Hohmann, Javier Echeverria, Hung-Rong Yen, Aiping Lu, Michael Heinrich

**Affiliations:** ^1^ Department of Biological Science, College of Science, Sungkyunkwan University, Samsung Advances Institute of Health Science and Technology (SAIHST), Suwon, South Korea; ^2^ Facultad de Medicina, Universidad San Sebastián, Concepción, Chile; ^3^ Research and Development Service, Bay Pines VA Healthcare System, Bay Pines, FL, United States; ^4^ Department of Pharmacognosy, University of Szeged, Szeged, Hungary; ^5^ Departamento de Ciencias del Ambiente, Facultad de Química y Biología, Universidad de Santiago de Chile, Santiago, Chile; ^6^ Research Center for Traditional Chinese Medicine, China Medical University, Taichung, Taiwan; ^7^ School of Chinese Medicine, Hong Kong Baptist University, Kowloon, Hong Kong SAR, China; ^8^ Research Group “Pharmacognosy and Phytotherapy”, UCL School of Pharmacy, Univ.London, London, United Kingdom

**Keywords:** insights in ethnopharmacology: 2021, ethnomedicine, future perspectives, phytochemicals, therapeutic strategies

Over 20 years after the start of the new millennium, ethnopharmacology clearly is making essential contribution to understanding medicines and their pharmacology, but at the same time science in general faces new and crucial challenges, not the least with regards to climate change and the need to treat existing and emerging major diseases. Human beings have always been faced with the unexpected. As humans we have always lacked experience, have been doubtful or hopeful, disappointed or forward looking. These developments have accelerated since the modernization, “Westernization” or globalisation of societies. We, today, all accept a historic milestone dividing the developments–in philosophical and scientific terms–into pre-modern and modern times, considering René Descartes (1596–1650) as the father of modern philosophy (Rodis-Lewis, 1998) and Galileo Galilei (1564–1642), as the father of science (Brodrick, 1965). Since Charles Darwin’s (1809–1882) revolutionary conceptualisation of biological evolution current biological thinking and practice including pharmacology has made tremendous contributions to our understanding of the natural world and humans’ place in it (Browne, 1995). Methods and technologies relevant for the pharmacological approaches are now stepping up to new levels of complexity focusing on evidence-based approaches in many areas including in understanding the metabolism, structure, interaction and combinational action of human cells, tissues and organisms and how this can be used to treat and prevent diseases.

Ethnopharmacology has gained a global reputation most notably in Asian countries as an approach which can contribute to such developments. Evidence for the efficacy, pharmacological effects and relative safety of traditional herbal medicine has resulted in advantages in treating human diseases even for indications, we had not considered some decades ago. To meet the challenges of globalising such local or traditional uses, ethnopharmacology has to embrace the standards of pharmacological research practice. If pharmacokinetic safety and pharmacodynamic effects are guaranteed, a wider therapeutic use can be endorsed and this will most likely depend on the competent regulatory agencies of the nearly 200 countries globally. Various phytotherapeutic preparations have been known to treat many human diseases in the forms of decoction, extracts, or powder (Heinrich et al., 2023). Its future direction therefore includes field, pharmacological, clinical and case studies of local and traditional medicines in clearly defining and quantifying metabolites relevant for the activity (both directly and indirectly).

The present topic highlights integrative approaches using different both classical and modern pharmaceutical preparations (including decoctions, pills, powders, and supercritical fluid extracts) targeting migraine, cholestatic hepatic injury, rheumatoid arthritis and lipid malfunction. Intractable diseases including ischemic stroke, hyperglycaemia, skin photo-ageing, gastrointestinal and respiratory tract infections have been highlighted by reviews. This Research Topic includes eleven independent publications of seven reviews and four original research papers, as below:

1) Crocins for ischemic stroke: a review of current evidence by Shahbaz et al. Crocins (CRs) and other metabolites from *Crocus sativus* L. (Saffron) are clinically effective for ischemic stroke and cerebral ischemia. Saffron and CR are effective in metabolic syndrome, depression, Alzheimer’s disease, neuro- and cardiovascular diseases by multiple mechanisms with mitochondrial apoptosis, NF-κB, S100 calcium-binding protein B, IL-6 and VEGF-A. Pharmacokinetically, CR is poorly bioavailable and conversion to crocetin allows translocation of the blood-brain barrier. While this certainly is a very interesting botanical drug, further research will need to focus on a larger scale sustainable production of the source material
2) Role of phytochemicals in skin photoprotection *via* regulation of Nrf2 by Chaiprasongsuk and Panich. The review highlights photoprotecting phytochemicals for skin *via* Nrf2. It focuses on natural products exerting skin protective and photoprotective effects mitigating ultraviolet radiation (UVR)-caused skin damage *via* nuclear factor erythroid 2-related factor 2 (Nrf2) including potential effect against photoaging and hyperpigmentation. The review highlighted phytochemicals-targeted Nrf2 in photoprotection of skin.
3) Phytochemistry in the Ethnopharmacology of North and Central America by Arnason et al. (review). This paper highlights phytochemicals of plants in North and Central America. New integrated approaches have been applied for metabolomic biomakers and synergist effects on phytochemicals, plant species and cultivars. Taking a geographical approach, this paper highlights the contribution of ethnopharmacological research in the context of a continent where more research on the local resources seems essential.
4) Approaches to decrease hyperglycemia by targeting impaired hepatic glucose homeostasis using medicinal plants by Mata-Torres et al. (review). It highlights pharmacological strategies using medicinal plants for preventing and treating hyperglycemia caused by hepatic insulin resistance. *Coreopsis tinctoria* Nutt., *Lithocarpus polystachyus* (Wall. ex A.DC.) Rehder and *Panax ginseng* C.A.Mey have been reviewed for effective glucose metabolism. Phenolic compounds and terpenoids are involved in the gluconeogenic pathway.
5) Bupleuri radix for acute uncomplicated respiratory tract infection: a systematic review of randomized controlled trials by Yan et al. (review). This highlights efficacy, clinical effectiveness, and safety issues of the *Bupleuri radix* (Bupleurum spp.) on acute uncomplicated respiratory tract infections (ARTIs) covering literature indexed in English and Chinese databases since 2021. The authors have used the Cochrane Risk of Bias Tool 2.0. RevMan 5.4 software. Injection solutions, pills, and decoctions similarly exhibit effects on AURTI symptoms such as nasal discharge and cough, calling for antipyretic effect.
6) P*lectranthus ecklonii* Benth: a comprehensive review into its phytochemistry and exerted biological activities by Antão et al. This review highlights genus *Plectranthus* (Lamiaceae), which has anti-inflammatory and anti-microbial activities in gastrointestinal and respiratory-related diseases. Diterpenes, triterpenes, flavonoids, and hydroxycinnamic acids of *P. ecklonii* are discussed.
7) *Reynoutria japonica* Houtt. for acute respiratory tract infections in adults and children: a systematic review by Wang et al. *Reynoutria japonica* Houtt. [alsoknown as *Fallopia japonica* (Houtt.) Ronse Decr.)] has been highlighted due to heat clearing, blood and qi circulating, phlegm elimination, and cough relieving activities with resveratrol and glycosides. From databases obtained from randomized trials, the respiratory tract infections-effective agents have been searched.
8) Study on the chemical constituents and anti-migraine activity of supercritical CO_2_ extracts of *Zanthoxylum schinifolium* by Yuan et al. (original research). Supercritical CO_2_ extraction technology has been highlighted in phytochemical research, as applied to *Zanthoxylum schinifolium* Siebold & Zucc. (CO_2_-ZSE) as a condiment known to relieve migraine. CO_2_-ZSE decreased the levels of serum nitric oxide (NO), endothelin-1 (ET-1), calcitonin gene–related peptide (CGRP), IL-1β, NF-κB, p65, and IκBα, and increased 5-hydroxytryptamine (5-HT) level in migraine animals. Linalool reduces the frequency of scratching the head and the NO, ET-1, and CGRP levels with effective migraine therapy observed by vasomotor factors and the inflammatory pathway.
9) Integrating network analysis and metabolomics to reveal mechanism of Huaganjian decoction in treatment of cholestatic hepatic injury by Dong et al. (original research). Bioactive constituents with pharmacological mechanism have been approached, integrating networking and metabolomics in high cholestatic hepatic injury associated with PI3K/Akt/Nrf2 signaling/GSH synthesis. Huaganjian decoction (HGJD) was known for 300 years for liver diseases.
10) An integrative pharmacology model for decoding the underlying therapeutic mechanisms of Ermiao powder for rheumatoid arthritis by Wu et al. (original research). Erimiao powder composed of *Phellodendron amurense* Rupr. and *Atractylodes lancea* (Thunb.) DC. shows anti-rheumatoid arthritis effect through coordinated molecular mechanism,
11) Lipid metabolism and its mechanism triggered by supercritical CO_2_ extract of Adlay [*Coix lacryma-jobi* var. *ma-yuen* (Rom. Caill.) Stapf] bran in high-fat diet induced hyperlipidemic hamsters by Huang et al. (original research). This novel approach to extraction of job’s tears seeds highlights how improved technologies, as in this case supercritical fluid extraction form the basis for new economically relevant extracts. Adlay bran (AB-SCF) extracts improves lipid metabolism in high fat-diet-induced hyperglycemic animals. AB-SCF extract prevents body weight gains and improves serum TG, TC, LDL-C and HDL-C levels as well as cardiovascular risk, lipid peroxidation, cholesterol metabolism and bile acid excretion via action on lipoprotein lipase, AMPK, p-AMPK and fatty acid synthase. Lipids (linoleic acid and oleic acid) and non-lipid components of 3-O-(trans-4-feruloyl)-β-sitostanol, 3-O-(*cis*-4-feruloyl)-β-sitostanol, and β-sitosterol are synergistically active.

Ethnopharmacological research has become increasingly valuable in the development of botanical products and their bioactive phytochemicals as novel and effective preventive and therapeutic strategies for various diseases including genetic intractable diseases and environmental intractable diseases as well cosmetic products preventing photo-caused skin damage and photoaging-based dermal problems including pathological or non-pathological hyperpigmentation.

Similarly, many efforts focus on preventive interventions including the use of food supplements. All this offers insights into medicinal and ethnopharmacological potential for developing novel and effective therapeutic agents. Outputs from ethnopharmacological research have become much more widely recognised as source candidates of active phytochemicals preventive and therapeutic strategies. The biological activity of isolated metabolites also justifies the increased interest in multiple herbal species, keeps the spotlight on individual metabolites or combinatory drug therapy. The widespread analyses need to be assessed scientifically to better understand the individual metabolites and safeties of local and traditional medicines (Silveira et al., 2020; Kim, 2021; Park et al., 2021). Indeed, complementary and alternative medicine has been highlighted to prevent and treat intractable diseases. These are considered as a complementary supplement for functional foods or combined botanical drugs in the near future, although these discoveries remain to be confirmed and applicable by reasonable clinical trials. In future perspective, systemic evaluation of the effectiveness and safety of local and traditional medicines are important. Using eleven case studies, this Research Topic highlights the current state-of-the-art in ethnopharmacology. We as editors also want to encourage researchers to more systematically embrace the huge challenges in the field of medicinal plant research and their application. Sustainable sourcing (including the dramatic challenges of climate change), equitable benefits of the primary producers, a better understanding of the clinical efficacy of chemically well-defined extracts, the safety of these preparations and last but not least the need for truly transdisciplinary approaches in ethnopharmacology all are themes we as scholars need to tackle and contribute to their solution.
